# Genetic Diversity of *Stratiotes aloides* L. (Hydrocharitaceae) Stands across Europe

**DOI:** 10.3390/plants10050863

**Published:** 2021-04-25

**Authors:** Barbara Turner, Steffen Hameister, Andreas Hudler, Karl-Georg Bernhardt

**Affiliations:** 1 Department of Integrative Biology and Biodiversity Research, University of Natural Resources and Life Sciences, Gregor-Mendel-Straße 33, 1180 Vienna, Austria; sthameister@hotmail.com (S.H.); karl-georg.bernhardt@boku.ac.at (K.-G.B.); 2 Tiroler Umweltanwaltschaft, Meranerstraße 5/III, 6020 Innsbruck, Austria; a.hudler@tiroler-umweltanwaltschaft.gv.at

**Keywords:** AFLP, conservation, genetic diversity, river systems, *Stratiotes aloides*, wetland habitats

## Abstract

Intense land use and river regulations have led to the destruction of wetland habitats in the past 150 years. One plant that is affected by the reduction in appropriate habitats is the macrophyte *Stratiotes aloides* which has become rare in several areas. The preservation of genetic diversity within a species is a prerequisite for survival under changing environmental conditions. To evaluate the level of genetic diversity within and among populations of *Stratiotes aloides*, we investigated samples from waterbodies across Europe using AFLP. Low genetic diversity among samples from the same population was found, proving that stands consist of few clones which propagate clonally. Nevertheless, most populations showed differences compared to other populations indicating that there is genetic diversity within the species. The analyzed samples formed two groups in STRUCTURE analyses. The two groups can be further subdivided and mainly follow the major river systems. For conserving the genetic diversity of *Stratiotes aloides*, it would thus be preferable to focus on conserving individuals from many different populations rather than conserving selected populations with a higher number of individuals per population. For reintroductions, samples from the same river system could serve as founder individuals.

## 1. Introduction

The monotypic genus *Stratiotes* includes the sole living species *S. aloides* L. (water soldier) and is a member of the Hydrocharitaceae which belong to the order Alismatales within monocots. During the Tertiary and Quaternary periods, there were up to twenty different species of the genus *Stratiotes* in Europe and Asia [1] (and references therein). The free-floating aquatic macrophyte is perennial with leaves up to 40 cm long and 4 cm wide which are arranged in rosettes. Depending on the season, the plants are emerged or submerged [2]. During the vegetative and reproductive period of a year, the plants are mostly emergent with the rhizoids free in the water or loosely attached to the soil. In autumn, the plant submerges in order to overwinter at the bottom of the water until the following spring [2]. Besides sexual reproduction, the dioecious plants also propagate via vegetative organs (turions and offshoots). Since vegetative reproduction is much more common in *S. aloides*, stands in one waterbody are often formed by clonal individuals of the same sex [2,3]. As long as individuals from different sexes are not transferred from one waterbody to another by floods and high waters, sexual reproduction is very rare. As a typical flood plain species, it inhabits slow-moving or stagnant waters such as ponds, canals, ditches and oxbow waters where it often dominates macrophyte communities [1]. Stands of water soldiers are frequently inhabited by macroarthropod fauna of which several species are of conservation concern [4,5,6]. *Stratiotes aloides* is distributed from northern Middle Europe in the West to Siberia in the East.

Wetlands are among the most endangered habitats in Central Europe and at the same time, among the hotspots of European biodiversity [7,8]. Back waters are part of natural flood plains. They are independent habitat types with a special flora and fauna. Natural backwaters are caused by the dynamics of the watercourses, which cause seasonal fluctuations of the water level and thus, a temporary regional flood. Today, those dynamics no longer exist in the low- and high-water areas of our modern cultural landscape. Human settlement in floodplains, river straightening, power plant construction and other land uses have led to the systematic destruction of these habitats since the end of the 19th century [9]. Due to anthropogenic influences, there has been an increasing decline, since wetlands have been drained and replaced by grassland [10]. Natural back-waters are endangered by sinking groundwater tables and a lack of flow dynamics [9]. Not only have the habitats themselves been destroyed, but water quality has also decreased, especially due to the increase in nitrogen and nitrates, and has led to a further decrease in the biodiversity of wetland habitats [11,12]. Additionally, wrong management such as clearings of fish-ponds and ditches [3,13] leads to a decrease in water soldier populations. Due to the reduction in appropriate habitat, *S. aloides* has started to decline and is extinct at its southern and western distribution range [14] (and references therein). Apart from the already mentioned threats for wetland habitats and the biodiversity within them, introduced alien species also have to be mentioned as a severe threat to biodiversity in wetland habitats [15,16].

When *Stratiotes* waters are regularly flooded, the drifting away of parts of the population results in a transfer of plants to other areas and thus, to a genetic transfer and exchange between populations. Due to river regulations, flooding in riparian landscapes has decreased significantly. Only through extreme floods might it still be possible for *Stratiotes* to colonize new habitats via water ways [3]. Besides flowing water, vectors such as water birds play an important role in the dispersal of macrophytes (e.g., [17]). Although no study to our knowledge has directly investigated the dispersal of *Stratiotes* by birds, several authors mention the possibility of birds as dispersal vectors for *Stratiotes* [1,2,18]. Especially in regions such as central and eastern Europe, western Europe and secondary ranges in North America, where *Stratiotes* is mainly found in lakes and ponds with no water ways connecting these waterbodies, dispersal by birds seems to be likely. While vegetative parts seem to be too large to be transported by birds, seeds, if present, could possibly be dispersed endo- as well as exo-zoochorically, by birds [18]. However, independent genetic exchange through the transfer of individuals is unlikely in the regulated river areas of Europe. For example, the Austrian water soldier stocks are up to 55 km apart. Due to this geographical isolation, sexual reproduction between populations is no longer possible, because *Stratiotes* needs a pollination distance of less than one kilometer [19]. The maintenance of an evolutionary reproductive community, given through sexual reproduction or through the penetration of daughter individuals into other areas, and thus, the preservation of genetic diversity within and between the water soldier populations, is a prerequisite for the survival of the *Stratiotes* populations in changing environmental conditions [11,20]. Since *Stratiotes* reproduces clonally for the most part and the possibilities of gene flow through sexual reproduction and transfer of individuals are limited, it is assumed that there is a reduction in the genetic diversity of the species [21]. Knowledge about genetic diversity within a species and between populations of a species is necessary for in situ and ex situ conservation and following conservation concepts [22].

Here, in this study, we aim to investigate the genetic diversity of *Stratiotes aloides* populations across Europe to obtain insights into the circumference of the genepool of the species. These results could be helpful to find answers to conservation issues such as status of a population in a particular locality or possible source populations for recolonizations in habitats where *Stratiotes aloides* has already become extinct.

## 2. Results

After excluding 102 replicates, the final matrix used for analyses contained 345 individuals and 1320 fragments.

Results based on uncorrected p-distances and Hamming distances gave the same clustering patterns in neighbor-joining (NJ) dendrograms and principal coordinate analysis (PCoA). The same was found for the pair of Dice distances and Jaccard distances. Therefore, we used only results based on uncorrected p-distances and Dice distances for further analyses.

### 2.1. Neighbor Joining

**Figure 1:**
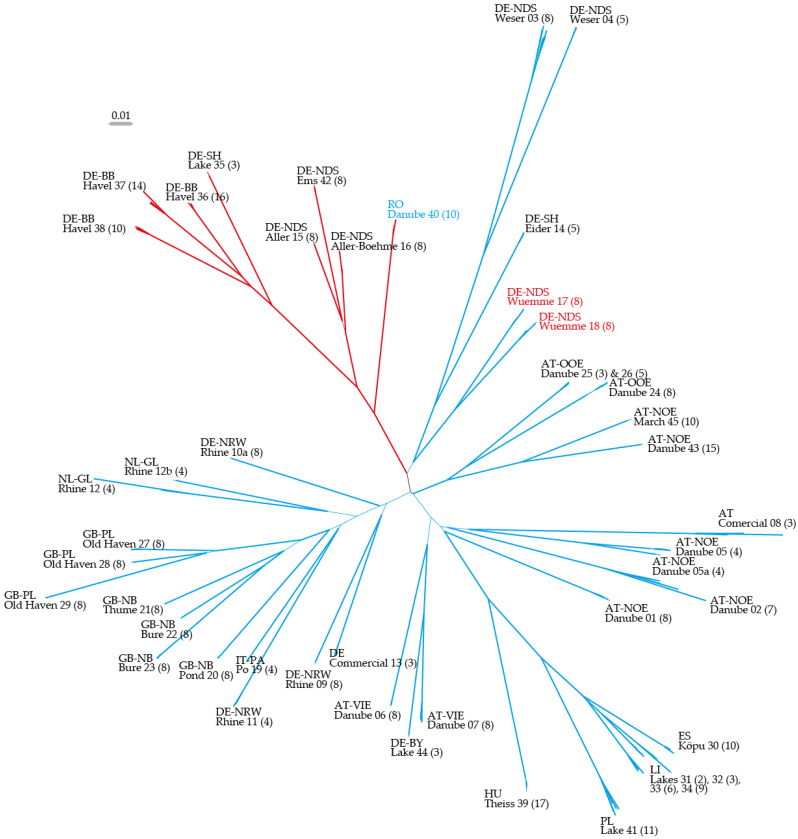
Unrooted NJ dendrogram based on Dice distances. Colors according to STRUCTURE results (K = 2); red—central highlands and plains; blue—rest of the sample regions.

NJ dendrograms based on uncorrected p and Dice distances both showed a star-like shape with a backbone of relative short branches lacking bootstrap support greater than 75% (Figure 1). They differed slightly in clustering patterns, but all of the differing branching patterns did not receive high bootstrap support in either of the two analyses. The groups found in STRUCTURE analyses and in PCoA are partly found in the NJ dendrograms. The two groups “Baltic + Hungary” (BH) and “Central European Highlands and plains 1 + Romania” (CER) based on STRUCTURE analyses (K = 5) are supported with high bootstrap values in the NJ dendrograms (BH: 99.7% in Dice, 89.8% in uncorrected p; CER: 99.9% in Dice and uncorrected p). Here, we present only unrooted trees due to the low resolution of their backbone.

### 2.2. STRUCTURE

**Figure 2:**
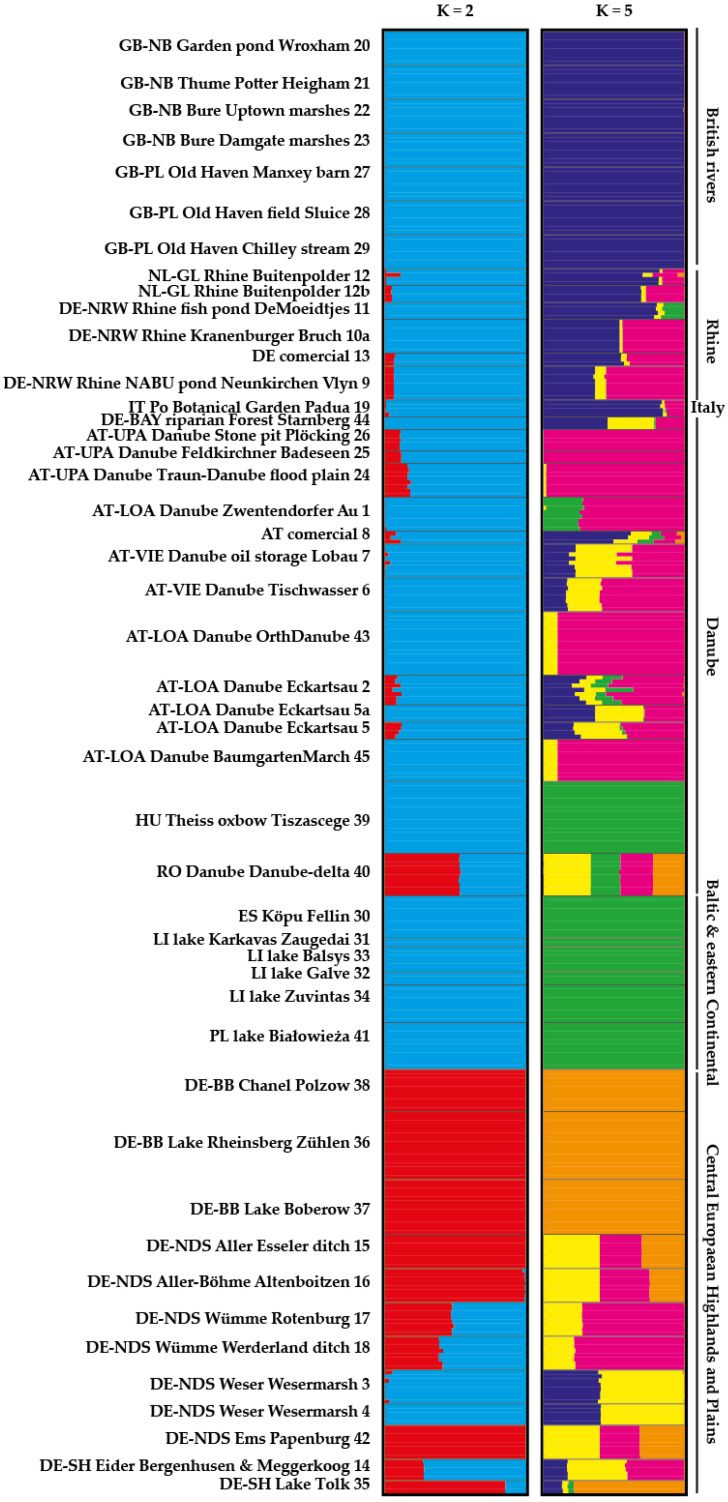
STRUCTURE results (K = 2 and K = 5) of the whole dataset.

STRUCTURE analysis gave the highest value of ΔK for K = 3 plus a few other suboptimal K values (Figure S1a) in the analysis of the reduced dataset (one or two representative individuals per population). However, the latter contained clusters with negligible membership (“empty” clusters). Visualization of K = 45 based on the STRUCTURE results (reduced dataset) showed six clusters which are subsets of the clusters in K = 3 (Figure S1b). STRUCTURE analysis of the whole dataset gave the highest value of ΔK for K = 2 and a suboptimum for K = 5 (Figure S2). The fastSTRUCTURE results gave a model complexity that maximizes marginal likelihood of 33 (this corresponds to the highest value of ΔK in STRUCTURE). These 33 potential clusters circumscribe mainly the sampling localities/populations with some populations being fused together (Figure S3). However, both NJ and PCoA analyses based on different distance measures are in correlation with clustering based on STRUCTURE rather than those based on fastSTRUCTURE. The main grouping found in STRUCTURE analyses (K = 2) and PCoA separates the samples into two groups and some admixed individuals (Figure 2). Group 1 includes only samples, but not all, from waterbodies within the catchment of the central European highlands and plains (populations 15; 16; 35–38; 42). The populations from Romania (Danube) and rivers Wümme and Eider (central European highlands and plains) appeared admixed. The rest of the samples (British rivers, Rhine, Danube, Baltic and eastern–central, two populations from the central European highlands and plains) forms the second group. A deeper look at the clustering patterns in PCoA and STRUCTURE analyses shows that both main groups can be further subdivided. Within the group of the central European highlands and plains (CE), samples from the Havel lowering in Brandenburg form a cluster together with the individuals from Lake Tolk in Schleswig-Holstein, which forms the core CE group. Samples from rivers Aller and Ems in Lower Saxony (CE-AE) appear to be admixed between the core CE group, Danube and Weser. Individuals from rivers Wümme and Eider appear to be related to populations from the Danube region and Weser. The two populations from the river Weser form an individual group with around 1/3 the impact of the British and Rhine populations. Within the second, much bigger group, samples from British rivers form a group as well as the samples from the Baltic and eastern central rivers together with the population from the Theiss river in Hungary (BH). Samples from Danube waters in Austria form a group with more or less impact from the Rhine, Weser and BH. Individuals from waterbodies along the Rhine river are a mixture between the British and the Danube genepools. The same was found for the population from the Botanical Garden of the University in Padua, which should originally be from the Po river. The only population that cannot be assigned to any of the groups is the population from the Danube estuary in Romania because this population shows impacts from Weser, Baltic, CE and Danube genepools.

### 2.3. Principal Coordinate Analyses

**Figure 3:**
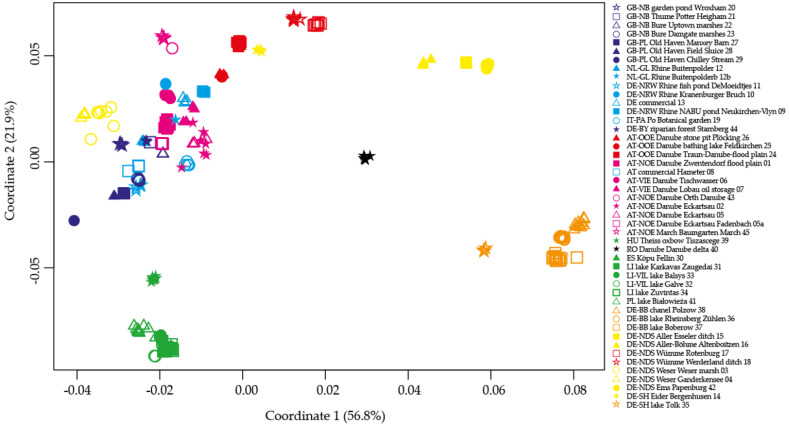
PCoA based on uncorrected p-distances.

Principal coordinate analyses based on the two distance methods (uncorrected p and Dice) gave very similar clustering patterns, with uncorrected p-distances showing a higher total sum of coordinates (Table S1). The first coordinate (uncorrected p: 57%; Dice: 49%) separates the two main groups found in STRUCTURE analyses from each other with the admixed samples in between the two groups. The second coordinate (uncorrected p: 22%; Dice: 25%) separates the two main groups into two subgroups each. (Figure 3). The CE group is separated into the core CE group and the Aller-Ems group (CE–AE). The second group is separated into the BH group and a continuum of samples from British waterbodies, Rhine, Danube, Po and Weser. Among the PCoA based on pairwise *F*_ST_ distances from hierarchical AMOVAs, those based on groupings according to the STRUCTURE results gave the highest values, and also gave the highest values over all PCoA.

### 2.4. AMOVA and Population Statistics

In order to quantify the amount of genetic variation between populations, we have performed AMOVAs. When keeping all sampling sites as separate populations, the analysis showed 97% of the molecular variance occurred between the populations and *F*_ST_ value of 0.97 (Table S1). If populations are assigned according to STRUCTURE results (K = 2), the amount of molecular variance between populations drops to 33% and the *F*_ST_ value to 0.33. To investigate alternative groupings apart from the one based on STRUCTURE results, we also conducted AMOVAs for groupings based on fastSTRUCTURE results and river systems. Both of these groupings gave higher *F*_ST_ values than the grouping based on STRUCTURE results (Table S1). Average gene diversity over loci in non-hierarchical AMOVA was 0.214; in hierarchical AMOVA based on populations, average gene diversity varied between 0.047 within the commercial samples from Stauden Hameter (population 8) and 0.000 within samples from Potter Heigham (population 21) and Chilley Stream (population 29).

Nei’s H-value (unbiased expected heterozygosity) was estimated with uHe = 0.213 in the overall analysis of all samples together. Analysis of separate populations gave the highest Nei’s H-value of H = 0.031 for the commercial samples from Austria (Stauden Hameter population 8) and the lowest value (H = 0.000) for populations from the UK (Potter Heigham, population 21; and Chilley Stream, population 29). Shannon’s index was estimated to be I = 0.344 in the overall analysis of all samples together. In the analysis of separate populations, the highest and lowest values were found in the same populations for Nei’s H (for details, see Table S2).

### 2.5. Mantel Tests

Mantel tests based on different distance matrices showed between 2.3 and 100% correlation (R^2^) among the tested pairs of matrices (Table S3). The highest correlations were found between matrices based on uncorrected p-values, Dice distances and binary distances calculated with GenAlEx (r = 0.95–1). The lowest correlations were observed between the matrices based on the genetic data and the matrix containing the geographical data (r = 0.15–0.31), indicating that there is no or only little correlation between the geographic distance and genetic distance of the samples in our dataset. The only pair of datasets where a correlation (r = 0.64) between geographic distance and genetic-based AMOVA distances was observed is the pair of geographic distances and AMOVA distances based on grouping according to river systems.

## 3. Discussion

Here, in this study, we examined the genetic diversity of *Stratiotes aloides* populations from different water systems across Europe. As this species propagates mainly vegetatively, genetic diversity within populations is expected to be low. Due to missing connections (river regulations and lack of flooding) between the water systems, genetic diversity between populations is expected to be high.

Indeed, we did find a high *F*_ST_ value (0.97) when analyzing the populations separately which shows a high level of genetic differences between the populations and a very low level of genetic differences within the populations, indicating that the populations consist mainly of clones of few genetically different individuals. Considering that the populations propagate vegetatively, and that today, there is no gene flow between the populations via transfer of individuals from one population to another, the relations between the populations could show historical connections between populations. This explains why no, or if only a medium, correlation between geographic distance and genetic distance of the populations is found. The observed correlation between geographic distances and genetic distances based on AMOVA grouped by water bodies has to be viewed with some precaution, as the grouping based on water bodies is, of course, a geography-related grouping and will, therefore, already have a slight bias towards a stronger correlation. Nevertheless, we can still see that there is some correlation between geographic distance and genetic distance when we look at it at the level of waterbodies. The populations from waterbodies of the central European highlands and plains in particular have a genepool which is different from the genepool shared by populations from other regions in Europe. However, it looks like that geneflow between populations has occurred. The fact that water soldier populations from waterbodies along the Rhine seem to be a mixture of genepools from British and Danube genepools might be explained by the historic watercourse of the river Rhine with pervious headwaters of the Danube being directed to the Rhine and a common delta of the Rhine and Thames [23]. A second hypothesis for the connections between populations from British rivers, the Rhine and Italian rivers is long distance dispersal of seeds by migrating water birds (for an example of migration routes of ducks across Europe, see [24]). The connections between the populations from Poland, Baltic countries and the river Theiss in Hungary might also be explained by transfer of plant material by birds [25]. There are not much data available about the dispersal of water soldier fruits by animals, but Efremov et al. [1] and Orsenigo et al. [14] give an overview of the current knowledge of dispersal of *Stratiotes* and Cook and Urmi-König [2] as well as Forbes [18] mention birds as possible dispersals vectors. Summed up, animals can disperse *Stratiotes aloides* fruits exo- and endo-zoochorically and if they are migrating over longer distances, seeds and thus genetic information can be transferred between localities. A further point that has to be kept in mind, when investigating relationships among European water soldier populations, is the fact that *Stratiotes aloides* has a long history as an ornamental plant [1,2]. Unexpected and probably by natural means, difficult to explain relationships between populations could be the result of human-mediated transfer of plant material. As the earliest known fossils of *Stratiotes aloides* date back around 45 million years [26], the observed groups could be the result of repeated glaciation and deglaciation events in Europe [1]. Summing up, we found the investigated populations of *Stratiotes aloides* across Europe to form two main groups which can be further subdivided. Roughly, the two groups can be referred to as a central northern Europe-group (CE) and a western–southern–eastern Europe group.

Previous studies of *Stratiotes aloides* across its distributional range showed a much higher level of genetic diversity within the examined populations [14]. As the sampling regions of the study of Orsenigo et al. [14] are not the same as in our study, the main clustering structures of European populations cannot be fully compared. However, clustering of samples from the Rhine in The Netherlands and the Po in Italy, together with some similarities to populations from the Danube in Bavaria, was observed in both studies. Comparable genetic differences within and between populations of dioecious Hydrocharitaceae were found in *Ottelia acuminata* where high levels of genetic differences between the investigated populations were found, but little diversity within the populations [27].

All still present-day populations of *Stratiotes aloides* found in Europe are remnants of much larger and connected populations. For example, in the Danube flood plains around Vienna, *Stratiotes aloides* was still very common by the mid-19th century, around 100 years later, this species was already mentioned to be rare [28] (and references therein). This example shows that previous large and vital populations became rare and fragmented within the last 150 years.

One possible hypothesis for explaining the differentiation of the samples into two groups could be differences in ploidy level. Orsenigo et al. [14] mention that different ploidy levels (diploid and tetraploid) were observed in *Stratiotes aloides*. Unfortunately, the material available for our study was not appropriate for chromosome counts and genome size measurements.

Summing up the results and viewing them in light of conservation issues, we can conclude that for conserving the genetic diversity of *Stratiotes aloides*, it would be preferable to focus on conserving individuals from many different populations all over its distributional range, rather than conserving selected populations with a higher number of individuals per population. For reintroductions, samples from closely located populations, or at least from populations from the same river system, could serve as founder individuals. As sexual reproduction is rare in natural populations, ex situ collections of samples of both sexes might be established to facilitate sexual reproduction and thus, maintain or even slightly increase genetic diversity in *Stratiotes aloides*. Apart from protecting and conserving *Stratiotes aloides* as a species, protection of the species as a habitat for fauna species such as the dragonfly *Aeshna viridis* [29] and the black tern *Chlidonias niger* [30], which fully or at least mainly depend on *Stratiotes* [14], is important.

## 4. Materials and Methods

### 4.1. Material

Material was continuously collected between 2012 and 2018 all over Europe wherever populations of *Stratiotes* were found. Depending on the size of the populations and on the accessibility of the individuals, between 5 and 20 individuals per population were sampled. Wherever possible, individuals from the whole waterbody were collected (e.g., North and South shore, etc.). Short (approx. 5–7 cm) pieces of leaves were collected and immediately dried in silica gel. From several populations, herbarium specimens were collected and deposited in the herbarium of the University of Natural Resources and Life Sciences, Vienna (WHB). Herbarium accession numbers are indicated in the table of accessions (Table 1). In total, we included 345 individuals from 46 populations into the final analysis. As previous studies [14] showed that there is no detectable genetic difference between the two sexes, we did not pay attention to the sex of the collected individuals (for some populations, information about sex is available and can be requested from the authors).

### 4.2. DNA Extraction

DNA was extracted from 20 mg silica gel dried leaf material per individual. The material was ground into a fine powder in 2 mL tubes together with three glass beads in a Tissue-Lyser (Qiagen, Germantown, MD, USA) with 20 s^−1^ for 5 min. Extraction of DNA was performed via QIAcube (Qiagen) using the DNeasy Plant Mini Kit (Qiagen), mainly according to the manufacturer’s protocol. Exceptions were elution of DNA from the columns, which was performed with two steps of 50 µL of elution buffer each. RNA was digested after DNA extraction using 1 µg RNAse A and incubated at 37 °C for 30 min.

Quality control of the DNA extracts was performed photometrically using a NanoDrop 2000 spectrometer. To check RNA digestion, samples were loaded on a 0.8% agarose gel.

### 4.3. AFLP

All DNA extracts that met the quality criteria were adjusted to 100 ng/µL and used for AFLP fingerprinting. Preparation of AFLP samples mainly followed the original protocol [32] with slight modifications.

Restriction of genomic DNA with two restriction enzymes (*Eco*R I and *Mse* I) and ligation of double-stranded adaptors to the resulting restricted fragments were performed in one step in a thermal cycler (37 °C for 2 h followed by a hold at 10 °C). Reactions comprised 1.1 μL 10× T4 DNA ligase buffer (Promega, Madison, WI, USA), 1.1 μL 0.5 M NaCl, 0.55 μL BSA (1 mg/mL; New England BioLabs, Ipswich, MA, USA), 50 μM *Mse* I adaptors (genXpress, Selangor, Malaysia), 5 μM *Eco*R I adaptors (genXpress), 1 U *Mse* I restriction endonuclease (New England BioLabs, Ipswich, MA, USA), 5 U *Eco*R I restriction endonuclease (New England BioLabs), 67 U T4 DNA ligase (Promega), and 5.5 μL DNA (100 ng/µL) and were made up to a total volume of 11 μL with sterile water. Ligated DNA fragments were diluted 10-fold with TE buffer (0.1%). Preselective amplification reactions contained 1 μL 10× polymerase buffer (ThermoFisher Scientific, MA, Waltham, USA), 0.2 U DreamTaq DNA polymerase (ThermoFisher Scientific, Waltham, MA, USA), 0.1 μL dNTPs (0.25 µM; ThermoFisher Scientific), 0.55 μL preselective primer pairs (*Eco*R I -A and *Mse* I -C, each 5 μM; Sigma, St. Louis, MO, USA), 2 μL diluted restriction ligation product, and were brought to a total volume of 10 μL with sterile water. Amplification was carried out with the following profile: 2 min at 72 °C, 20 cycles of 20 s denaturing at 94 °C, 30 s annealing at 56 °C, 2 min extension at 72 °C, and a final extension step for 30 min at 60 °C. The preselective PCR products were diluted 10-fold with sterile water. Reactions for selective amplification contained 1 μL 10× Polymerase buffer (ThermoFisher Scientific), 0.1 U DreamTaq DNA polymerase (ThermoFisher Scientific), 0.1 μL dNTPs (0.25 µM; ThermoFisher Scientific), 0.55 μL *Mse* I-primer (5 μM; Sigma), 0.55 μL *Eco*R I-primer (1 μM; Sigma), and 2 μL diluted preselective amplification product and were brought to a total volume of 10 μL with sterile water. They were carried out in with the following profile: 2 min at 94 °C, 9 cycles of 10 s at 94 °C, 30 s at 65–57 °C (reducing the temperature at 1 °C per cycle), 2 min at 72 °C, 25 cycles of 10 s at 94 °C, 30 s at 56 °C, 2 min at 72 °C and a final extension for 30 min at 60 °C. All PCR steps and incubations were carried out in an Eppendorf Mastercycler Gradient. The selective PCR products were purified using Sephadex G-50 Superfine (Cytiva Life-Sciences, Marlborough, MA, USA) applied to a MultiScreen-HV 96-Well Plate (Millipore) in three steps of 200 μL each (5× *g* sephadex in 60 mL 1× TE-buffer) and settled at 750× *g* (1, 1 and 5 min, respectively). The same speed was used for centrifugation of the samples (selective PCR products: 3.7 µL of NED, 3.15 µL of FAM and 4.3 µL of VIC), again for 5 min. One microliter of the eluate was combined with 15 μL HiDi and 0.25 μL LIZ 600 (Applied Biosystems, ThermoFisher Scientific, Waltham, MA, USA) and denatured for 3 min at 95 °C before running them on a capillary sequencer (GA3500, Applied Biosystems, ThermoFisher Scientific).

The selective primer pairs (FAM-*Eco*RI-ACT/*Mse*I-CTA, VIC-*Eco*RI-ACG/*Mse*I-CTA and NED-*Eco*RI-ACC/*Mse*I-CTA) were chosen after testing seven different primer combinations in a preliminary test. The selected primer combinations generated clear and not too many bands, thus decreasing the risk of fragments co-migrating by chance, but still with sufficient variability to distinguish the samples.

Reproducibility was checked by repeating ca. 23% of the samples.

### 4.4. Scoring and Phylogenetic Analysis

Sizing and scoring of the data were performed with GeneMarker v2.4.0 (SoftGenetics, State College, PA, USA). After pre-analysis using default settings, sizing profiles of all samples were checked and where necessary, manually corrected. Most of these corrections concerned the 20 bp peak of the size standard. These peaks were often not correctly recognized by the GeneMarker program. High-quality sizing profiles (score > 90) were obtained for all samples. A panel of scorable fragments was established for each primer combination, and fragments between 30 and 600 bp were scored. The relative fluorescent unit (RFU) threshold was set at 40. Automatic scoring was conducted using Local Southern peak call, peak saturation, baseline subtraction, spike removal, pull up correction, and a stutter peak filter of 5% [33]. The results were exported as a presence/absence matrix. The outcome of the automatic scoring was manually checked and corrected for errors. These errors mostly concerned peaks for which shape was atypical. In total, 447 samples corresponding to 345 individuals were scored. From 78 individuals, replicate samples were performed (between two and four replicates per individual). Peak shifts between different analyses dates of the same individuals were used to correct and align all fragment analyses over the whole timespan of the project. These corrections were performed manually and very carefully to avoid artefacts within the dataset. Most of these corrections were small shifts of the majority of peaks by one or two base pairs. For the final analyses, we ended up with 345 individuals, for which high-quality fragment profiles for all three primer combinations could be obtained.

All three primer combinations were combined in a single matrix and analyzed together. Different distance measures were tested for their power to resolve relationships with our dataset. Distance matrixes were calculated in PAUP* v4.0a167 [34] (Nei–Li distance) and SplitsTree v4.15.1 [35] (uncorrected p, Dice, Jaccard and Hamming). Phylogenetic relationships based on previously mentioned distance matrices were reconstructed using SplitsTree to create unrooted NJ dendrograms. To assess the robustness of branches, NJ-bootstrap (NJ-BS) analyses were performed using SplitsTree.

To visualize the pattern of genetic clustering of individuals and populations, we plotted principal coordinate analysis (PCoA) using the R packages “ecodist” [36] and “scatterplot3d” [37] based on an individual uncorrected p matrix, and, respectively, on AMOVA-derived pairwise *F*_ST_ distances calculated with Arlequin v3.5.2.2 [38].

To investigate further significant groupings of the included individuals, we used the programs STRUCTURE v.2.3.4 [39,40,41,42] and fastSTRUCTURE v 1.0 [43]. STRUCTURE was initially run for K = 1–50 with a subset of one or two individuals per population to keep analysis time in a reasonable frame. Based on those results, a second STRUCUTRE analysis with the full dataset (345 individuals) for K = 1–8 was run. We ran STRUCTURE with 10 replicates each and a model based on admixture and independent allelic frequencies, without considering information regarding sampling localities. Each run had 100,000 iterations with 10% additional burn in. The calculation of delta K (ΔK) [44] and preparation of the input file for Clumpp were performed with Harvester [45]. Production of a combined file from the ten replicates of the best K was performed using Clumpp v1.1.2 [46] with the Greedy search algorithm. The graphical representation of STRUCTURE results was prepared with Distruct v1.1 [47]. FastSTRUCTURE was ran with the full dataset for K = 1–50. The calculation of ΔK and graphical representation of results were performed with the functions “chooseK.py” and “distruct.py”, both implemented in the fastSTRUCTURE package.

Both non-hierarchical and hierarchical analyses of molecular variance (AMOVA) and calculations of population statistics were conducted using Arlequin v3.5.2.2 [38]. The Excel plugin GenAlEx v6.503 [48] was also used for calculating population statistics, AMOVAs, PCoA and Mantel tests. For hierarchical AMOVAs, groups have been defined based on different possible clustering according to populations (sampling locality), river systems (grouped according to Trockner et al. [31]) and STRUCTURE results. Mantel tests [49] were performed based on distance matrices calculated with SplitsTree, pairwise *F*_ST_ values from AMOVAs, binary distances calculated with GenAlEx and geographic distances (calculated with Geographic Distance Matrix Generator v 1.2.3; [50]). Calculations of Nei’s heterozygosity [51], Shannon’s information index [52] and percentage of polymorphic fragments was performed with GenAlEx.

## Figures and Tables

**Table 1:** Table of accessions.

Pop nr	River System ^1^	Country	Region	Location	Nr. Indivs.	Year	Collector	HBV Acc. Nr	Coordinates
8	commercial	Austria	commercial	Stauden Hameter	3	2012	(Hameister S)		N 48°17′03.03″ E 16°02′19.84″
1	Danube	Austria	Lower Austria	Floodplain Zwentendorf, Obere Placken	8	2012	Bernhardt K-G	56059 57014	N 48°22′14.00″ E 15°47′47.00″
2	Danube	Austria	Lower Austria	Eckartsau Fadenbach	7	2012	Hermann		N 48°08′03.96″ E 16°45′45.03″
5	Danube	Austria	Lower Austria	Eckartsau Fadenbach	4	2012	Bernhardt K-G		N 48°08′10.50″ E 16°46′52.80″
5a	Danube	Austria	Lower Austria	Eckartsau Fadenbach	4	2012	Hameister S		M 48°08′10.50″ E 16°46′52.80″
6	Danube	Austria	Vienna	Tischwasser	8	2012	Hameister S		N 48°11′34.49″ E 16°28′54.84″
7	Danube	Austria	Vienna	Oilstrage Lobau	8	2012	Hameister S		N 48°10′48.55″ E 16°29′47.30″
24	Danube	Austria	Upper Austria	Traun-Danube-floodplain	8	2013	Hameister S Hudler A		N 48°15′16.90″ E 14°23′18.20″
25	Danube	Austria	Upper Austria	Bathing lake Feldkirchen	3	2013	Hameister S Hudler A		N 48°19′41.20″ E 14°03′45.90″
26	Danube	Austria	Upper Austria	Stone-pit Plöcking	5	2013	Hameister S Hudler A		N 48°26′35.00″ E 14°00′14.20″
43	Danube	Austria	Lower Austria	Orth an der Donau/Steinafurt	15	2015	Lapin K		N 48°08′31.90″ E 16°41′03.70″
45	Danube	Austria	Lower Austria	Baumgarten ad March, Maritz South	10	2018	Gregor L		N 48°18′50.00″ E 16°53′12.00″
13	commercial	Germany	commercial	Holzum	1	2013	(Hameister S)		N 51°46′36.13″ E 06°24′12.77″
13	commercial	Germany	commercial	Stauden Förster	2	2013	(Hameister S)		N 52°25′10.68″ E 13°01′11.81″
9	Rhine	Germany	Nordrhein-Westfalen	NABU pond Neukirchen Vlyn	8	2013	Hameister S		N 51°26′48.60″ E 06°32′35.20″
10	Rhine	Germany	Nordrhein-Westfalen	Kranenburger Bruch	8	2013	Hameister S		N 51°47′14.20″ E 06°01′37.50″
11	Rhine	Germany	Nordrhein-Westfalen	Fishpond “De Moeidtjes”	4	2013	Hameister S		N 51°51′04.00″ E 06°10′14.60″
12	Rhine	Netherlands	Gelderland	Buitenpolder (Rhine back water)	8	2013	Hameister S		N 51°54′03.30″ E 06°03′39.90″
12b	Rhine	Netherlands	Gelderland	Buitenpolder (Rhine back water)	8	2013	Hameister S		N 51°54′03.50″ E 06°03′44.60″
14	CHP	Germany	Schleswig-Holstein	Eider-Bergenhusen	1	2013	Rasran L		N 54°22′06.41″ E 09°20′55.39″
14	CHP	Germany	Schleswig-Holstein	Eider-Bergenhusen NABU	2	2013	Rasran L		N 54°22′27.17″ E 09°19′24.49″
14	CHP	Germany	Schleswig-Holstein	Eider-Meggerkoog	2	2013	Rasran L		N 54°21′55.82″ E 09°22′47.45″
35	CHP	Germany	Schleswig-Holstein	Tolk-lake	3	2014	Rasran L		N 54°34′37.37″ E 09°37′37.37″
3	CHP	Germany	Niedersachsen	Weser marsh Bremen	8	2012	Bernhardt K-G		N 53°08′38.60″ E 08°39′24.60″
4	CHP	Germany	Niedersachsen	Ganderkensee-Werderland	5	2012	Hanke K		N 53°02′03.24″ E 08°32′33.52″
15	CHP	Germany	Niedersachsen	Aller, Esseler ditch	8	2013	Turner F		N 52°42′12.26″ E 09°37′30.91″
16	CHP	Germany	Niedersachsen	Aller (Böhme), Altenboitzen	8	2013	Turner F		N 52°48′49.25″ E 09°32′14.69″
17	CHP	Germany	Niedersachsen	Wümme, Rotenburg	8	2013	Turner F		N 53°05′49.86″ E 09°21′20.31″
18	CHP	Germany	Niedersachsen	Wümme, Werderland ditch	8	2013	Turner F	57455 57456	N 53°08′49.41″ E 08°38′25.49″
36	CHP	Germany	Brandenburg	Havel, Rheinsberg-Zühlen lake	16	2014	Grimm Oldorff S		N 53°03′55.44″ E 12°48′54.84″
37	CHP	Germany	Brandenburg	Havel, Boberow lake	13	2014	Grimm Oldorff S		N 53°10′57.11″ E 13°01′12.76″
38	CHP	Germany	Brandenburg	Havel, Chanel Polzow	10	2014	Grimm Oldorff S		N 53°07′03.17″ E 13°01′07.05″
42	CHP	Germany	Niedersachsen	Ems, Channel Papenburg	8	2015	Tremetsberger K	64641	N 53°04′27.15″ E 07°27′03.61″
44	Danube	Germany	Bavaria	Isar, Riparian forest Starnberg	3	2016	Bernhardt K-G	67455 67456	N 48°01′37.10″ E 11°23′32.60″
19	Italian	Italy		Po, Botanical Garden Padua	4	2013	Bernhard K-G, Hameister S		N 45°23′55.94″ E 11°52′50.69″
20	British	Great Britain	Norfolk	Garden pond, Norfolk Broads Wroxham	8	2013	Leaney B		N 52°42′21.02″ E 01°24′04.56″
21	British	Great Britain	Norfolk	Thume, Norfolk Broads, Potter Heigham	8	2013	Leaney B		N 52°42′14.34″ E 01°34′31.31″
22	British	Great Britain	Norfolk	Bure, Norfolk Broads, Uptown Marshes	8	2013	Leaney B		N 52°39′46.43″ E 01°32′31.98″
23	British	Great Britain	Norfolk	Bure, Norfolk Broads, Damgate Marshes	8	2013	Leaney B		N 52°37′54.15″ E 01°33′34.24″
27	British	Great Britain	East Sussex	Old haven, Pevensey Level, Manxey Barn	8	2013	Birch J		N 50°49′16.21″ E 00°21′3.45″
28	British	Great Britain	East Sussex	Old haven, Pevensey Level, Field Sluice	8	2013	Birch J		N 50°49′16.21″ E 00°21′03.45″
29	British	Great Britain	East Sussex	Old haven, Pevensey Level, Chilley Stream	8	2013	Birch J		N 50°49′16.21″ E 00°21′03.45″
30	BEC	Estonia	Vijandi	Köpu, Fellin	10	2014	Vellak K		N 58°20′09.00″ E 25°20′08.00″
31	BEC	Lithuania	Utena	Karkavas lake, Zaugedai	2	2014	Bernhardt K-G	62094	N 55°06′22.30″ E 25°40′08.78″
32	BEC	Lithuania	Vilnius	Galve lake	3	2014	Bernhardt K-G		N 54°39′00.20″ E 24°55′49.90″
33	BEC	Lithuania	Vilnius	Balsys lake	6	2014	Bernhardt K-G		N 54°47′01.50″ E 25°20′00.90″
34	BEC	Lithuania	Alytus	Zuvintas lake	9	2014	Bernhardt K-G	62091 62092 62093	N 54°27′26.40″ E 23°38′18.40″
39	Danube	Hungary	BH	Theiss oxbow, Tiszascege.	17	2014	Hameister S Oschatz		N 47°40′45.20″ E 20°59′01.90″
40	Danube	Romania	Tulcea	Danube-delta; E Tulcea. NE Murighiol.	10	2015	Bernhardt K-G	64193	N 45°08′38.10″ E 29°19′30.70″
41	BEC	Poland	Podlachien	Białowieża; Palace Park	11	2015	Wernisch MM	64088	N 52°42′05.32″ E 23°50′42.42″

^1^ Grouping of waterbodies into larger European river systems is based on classifications in Trockner et al. [31]; BEC: Baltic and Eastern Central; CHP: Central Highlands and Plains

## Data Availability

The data presented in this study are available as supplementary material File S1.

## Supplementary Materials

The following are available online at https://www.mdpi.com/article/10.3390/plants10050863/s1, Figure S1a: Delta K values of the reduced dataset (one or two individuals per population), Figure S1b: Visualization of STRUCUTRE results for K = 3 and K = 45 from the reduced dataset, Figure S2: Delta K values of the complete dataset, Figure S3: Visualization of fastSTRUCTURE results for K = 33, Table S1: Results of PCoA and AMOVA, Table S2: Overview of frequency values from population statistics, Table S3: Overview and comparison of Mantel test results. File S1: Data matrix containing AFLP data.
