# Supplementary material for: Genetic Diversity of Stratiotes aloides L. (Hydrocharitaceae) Stands across Europe
Source: Plants (Basel). 2021 Apr 25;10(5):863. doi: 10.3390/plants10050863 (PMC8145211; doi:10.3390/plants10050863)
Supplement: Supplementary file 1 [file plants-10-00863-s001.zip › Table S1.pdf]

	1st coordinate	2nd coordinate	3rd coordinate	Sum coordinates	F_{ST}	Variation within populations [%]	Variation among populations [%]	average gene diversity over loci
AMOVA STRUCTURE (K3)	99.2	0.8	0	100	0.35	65	35	
AMOVA STRUCTURE (K2)	91.8	9.2	0	100	0.33	67	33	
AMOVA water	83.5	12.0	3.1	98.6	0.34	66	34	
uncorrected P	56.8	21.9	8.5	87.2				
Hamming	56.8	21.9	8.5	87.2				
GenAlExDist	56.8	21.9	8.5	87.2				
Dice	48.7	25.4	10.1	84.2				
Jaccard	48.7	25.4	10.1	84.2				
AMOVA fastStructure (K33)	11.6	8.1	6.4	26.1	0.85	15	85	
AMOVA separate	5.4	4.8	4.1	14.3	0.97	3	97	0.04697

Supplementary Material 4: Results of PCOA and AMOVA
