# Supplementary material for: Genetic Diversity of Stratiotes aloides L. (Hydrocharitaceae) Stands across Europe
Source: Plants (Basel). 2021 Apr 25;10(5):863. doi: 10.3390/plants10050863 (PMC8145211; doi:10.3390/plants10050863)
Supplement: Supplementary file 1 [file plants-10-00863-s001.zip › Table S2.pdf]

Pop	Number of effective alleles (Ne)	Shannon' information-index (I)	Unbiased expected Heterozygosity (uHe)	Percentage of polymorphic loci
**Wroxham 20**	1.002	0.002	0.001	0.45%
**Potter Heigham 21**	1.000	0.000	0.000	0.00%
**Uptown marshes 22**	1.000	0.000	0.000	0.15%
**Damgate marshes 23**	1.002	0.002	0.001	0.53%
**Manxey Barn 27**	1.002	0.002	0.001	0.68%
**Field Sluice 28**	1.000	0.000	0.000	0.08%
**Chilley Stream 29**	1.000	0.000	0.000	0.00%
**Buitenpolder 12**	1.021	0.021	0.015	4.62%
**Buitenpolder 12b**	1.001	0.001	0.001	0.15%
**Fish-pond DeMoeidtjes 11**	1.008	0.007	0.005	1.21%
**Kranenburger Bruch 10a**	1.001	0.001	0.000	0.08%
**DE commercial 13**	1.007	0.006	0.005	0.91%
**Neukirchen Vlyn 09**	1.001	0.001	0.000	0.23%
**Padua 19**	1.007	0.006	0.004	0.98%
**Starnberg 44**	1.001	0.001	0.001	0.15%
**Stone-pit Plöcking 26**	1.001	0.001	0.001	0.30%
**Bathing-lake Feldkirchen 25**	1.003	0.002	0.002	0.38%
**Traun-Danube floodplain 24**	1.010	0.009	0.006	1.97%
**Zwentendorfer Au 01**	1.008	0.009	0.006	1.97%
**AT commercial 08**	1.044	0.039	0.031	7.05%
**Tischwasser 06**	1.002	0.002	0.001	0.45%
**Oilstorage Lobau 07**	1.007	0.008	0.005	1.97%
**Orth ad. Danube 43**	1.000	0.000	0.000	0.08%
**Eckartsau Fadenbach 02**	1.061	0.051	0.037	9.17%
**Eckartsau 05**	1.010	0.010	0.007	2.05%
**Eckartsau Fadenbach 05a**	1.001	0.001	0.001	0.38%
**Baumgarten ad. March 45**	1.001	0.001	0.001	0.38%
**Tiszascege 39**	1.004	0.004	0.003	1.06%
**Danube delta 40**	1.002	0.003	0.001	1.06%
**Köpu 30**	1.005	0.006	0.004	1.52%
**Zaugedai 31**	1.002	0.001	0.001	0.23%
**Balsys 33**	1.005	0.006	0.004	1.29%
**Galve 32**	1.001	0.001	0.000	0.08%
**Zuvintas 34**	1.009	0.010	0.007	2.88%
**Białowieża 41**	1.012	0.016	0.010	5.08%
**Polzow 38**	1.008	0.010	0.006	3.41%
**Zühlen Rheinsberg 36**	1.003	0.003	0.002	1.06%
**Boberow 37**	1.006	0.009	0.005	4.09%
**Esseler ditch 15**	1.000	0.001	0.000	0.30%
**Altenboitzen 16**	1.002	0.004	0.002	1.52%

**Rotenburg 17**	1.008	0.008	0.005	1.74%
**Werderland ditch 18**	1.012	0.010	0.007	1.82%
**Wesermarsch 03**	1.033	0.029	0.021	5.61%
**Wesermarsch 04**	1.003	0.003	0.002	0.61%
**Papenburg 42**	1.005	0.005	0.003	0.91%
**Eider 14**	1.006	0.006	0.004	1.06%
**Tolk lake 35**	1.002	0.002	0.002	0.38%
**All together**	1.360	0.344	0.213	94.09%

Supplementary Material 5: Overview of frequency values
