# Supplementary material for: Genetic Diversity of Stratiotes aloides L. (Hydrocharitaceae) Stands across Europe
Source: Plants (Basel). 2021 Apr 25;10(5):863. doi: 10.3390/plants10050863 (PMC8145211; doi:10.3390/plants10050863)
Supplement: Supplementary file 1 [file plants-10-00863-s001.zip › Table S3.pdf]

	r	P	R^2	R^2 %
Uncorrected P-dist	1.00	0.001	1.00	100
Uncorrected P-Dice	0.95	0.001	0.89	89.5
Dice-dist	0.95	0.001	0.89	89.5
geography-AMOVAwater	0.64	0.010	0.40	40.5
Dice-AMOVAindividual	0.56	0.001	0.32	31.8
Uncorrected P-AMOVAstruct	0.56	0.001	0.32	31.8
dist-AMOVAstruct	0.56	0.001	0.32	31.8
dist-AMOVAindividual	0.56	0.001	0.31	31.3
Uncorrected P-AMOVAindividual	0.56	0.001	0.31	31.3
Dice-AMOVAstruct	0.51	0.001	0.26	26.1
Dice-AMOVAwater	0.49	0.010	0.24	23.7
Uncorrected P-AMOVAwater	0.38	0.010	0.14	14.1
dist-AMOVAwater	0.38	0.010	0.14	14.1
Dice-geography	0.37	0.010	0.14	13.5
AMOVAstruct-AMOVAwater	0.36	0.010	0.13	13.2
AMOVAstruct-geography	0.31	0.010	0.10	9.7
AMOVAindividual-geography	0.26	0.010	0.07	7.0
Uncorrected P-geography	0.26	0.010	0.07	6.6
dist-geography	0.26	0.010	0.07	6.6
AMOVAindividual-AMOVAwater	0.25	0.010	0.06	6.0
AMOVAindividual-AMOVAstruct	0.18	0.001	0.03	3.3
AMOVApops-geography	0.15	0.030	0.02	2.3

Supplementary Material 6: Comparison of Mantel-tests
